# Pathophysiology of sepsis-induced myocardial dysfunction

**DOI:** 10.1186/s40779-016-0099-9

**Published:** 2016-09-27

**Authors:** Xiuxiu Lv, Huadong Wang

**Affiliations:** Department of Pathophysiology, Key Laboratory of State Administration of Traditional Chinese Medicine of the People’s Republic of China, School of Medicine, Jinan University, Guangzhou, Guangdong 510632 China

**Keywords:** Sepsis, Myocardial dysfunction, Pathogenesis, Clinical manifestation

## Abstract

Sepsis-induced myocardial dysfunction is a common complication in septic patients and is associated with increased mortality. In the clinical setting, it was once believed that myocardial dysfunction was not a major pathological process in the septic patients, at least in part, due to the unavailability of suitable clinical markers to assess intrinsic myocardial function during sepsis. Although sepsis-induced myocardial dysfunction has been studied in clinical and basic research for more than 30 years, its pathophysiology is not completely understood, and no specific therapies for this disorder exist. The purpose of this review is to summarize our current knowledge of sepsis-induced myocardial dysfunction with a special focus on pathogenesis and clinical characteristics.

## Background

Sepsis is a systemic deleterious host response to infection or injury resulting in severe sepsis and septic shock. It is a leading cause of morbidity and mortality in intensive care units [1, 2]. Although the hospital mortality of septic patients decreased from 37 to 30.8 % during the 2 years after the introduction of the Surviving Sepsis Campaign guidelines for the management of sepsis, mortality remains high [3]. In 2012, a global study of the burden of sepsis estimated that the case-fatality rate for patients with severe sepsis approaches 50 % [4].

The cardiovascular system plays an important role in the pathogenesis of sepsis. Over the last 50 years, a large number of studies have demonstrated that myocardial dysfunction is a common finding in septic patients, and approximately 50 % of septic patients exhibit signs of myocardial dysfunction. Nevertheless, the exact clinical significance of sepsis-induced myocardial dysfunction (SIMD) is still elusive. Because the heart, as only one part of the circulatory system, constantly responds to changing peripheral hemodynamics, it is difficult to distinguish between cardiac responses to alterations in preload, afterload or/and neurohumoral activity during sepsis and the direct influence of sepsis on the heart in the clinical setting [5, 6]. Recently, many clinical studies have suggested that myocardial dysfunction was associated with increased mortality in septic patients [7–9]. An animal experimental study showed that myocardial depression was present at the early stage of sepsis, and early myocardial functional changes could predict outcomes in septic animals [10]. In particular, using transgenic mice with cardiomyocyte-specific expression of a constitutively active PI3K isoform that protects myocardial function, Li and coworkers demonstrated, for the first time, a causal relationship between the maintenance of myocardial function and survival in sepsis. They found that cardiac specific activation of PI3K/Akt-dependent signaling significantly attenuated myocardial dysfunction and, in turn, improved survival in cecal ligation and puncture (CLP)-induced sepsis [11]. Therefore, fully understanding the pathogenesis of SIMD and seeking specific therapy will provide beneficial effects on outcomes in septic patients.

The aim of the present review is to discuss the pathophysiology of SIMD, with a special focus on its clinical characteristics and pathogenesis.

## The definition of SIMD

Although numerous studies have demonstrated evidence of cardiovascular impairments in patients with sepsis over the last 50 years, there is no universally accepted definition of SIMD [12]. The initial concept of SIMD came from the study by Parker and Parrillo et al. in 1984. They observed that 50 % of septic patients had a decreased initial left ventricle ejection fraction (EF) with increased mean end-systolic and end-diastolic volumes despite normal or elevated cardiac index found in all septic patients [13]. Since then, SIMD has been defined in numerous clinical investigations as a reversible decrease in EF of both ventricles, with ventricular dilation and less response to fluid resuscitation and catecholamines [14]. However, it is now well known that left ventricular EF is a load-dependent index that reflects the coupling between left ventricular afterload and contractility, rather than the intrinsic myocardial contractile function. During septic shock, although left ventricular intrinsic contractility is seriously impaired, left ventricular EF may be normal when the afterload is severely depressed [12, 15]. On the other hand, myocardial dysfunction has been demonstrated to be constant if determined by using the load-independent parameters of systolic and diastolic function in all animal experimental models of septic shock [16]. Thus, it has recently been suggested that SIMD can be defined as the intrinsic myocardial systolic and diastolic dysfunction of both the left and right sides of the heart induced by sepsis [12, 16].

## The clinical characteristics of SIMD

Hemodynamic alterations during sepsis have been investigated for 60 years. Early animal studies performed by Weil et al. in 1956 showed that an injection of endotoxin could cause a sudden decrease in venous return, arterial blood pressure and cardiac output (CO) with increased systemic vascular resistance (SVR), leading to animal death [17]. Clinical observations by Clowes and McLean et al. demonstrated that cardiovascular disturbances during septic shock included two distinct clinical pictures. One was an early hyperdynamic phase (warm shock) characterized by increased CO and decreased SVR, as well as warm and perfused skin; another was late hypodynamic phase (cold shock), in which SVR increased and CO decreased, resulting in tissue hypoperfusion, cool skin, organ failure and ultimate death [18, 19]. These findings led to the belief that patients with septic shock initially went through an early hyperdynamic phase and eventually either recovered or deteriorated into the hypodynamic phase and even death. However, these studies used central venous pressure (CVP) to reflect left ventricular end-diastolic volume and adequacy of resuscitation. In fact, we now know that CVP is not a reliable index of cardiac preload in septic patients. With the introduction of pulmonary artery catheters, which allow simultaneous measurement of both CO and pulmonary artery wedge pressure at the bedside, many studies have shown that septic shock patients or animals with adequate fluid resuscitation have only a persistent hyperdynamic state, which usually persists until death even in nonsurvivors, and the hypodynamic state is very likely due to an inadequate fluid resuscitation [20]. It is now generally accepted that these hemodynamic alterations during sepsis are due to decreased preload, reduced afterload, myocardial dysfunction, blood flow redistribution between organs and microcirculatory impairments [12].

### Changes in systolic and diastolic function

In 1984, Parker and Parrillo et al. provided the first valuable clue for the SIMD [13]. Using serial radionucleotide ventriculograms and simultaneous evaluation of CO by thermodilution, they demonstrated that 20 septic shock patients had a high CO and low SVR. Importantly, they further found that 13 patients who survived had a depressed left ventricular EF and acute left ventricular dilatation, which were sustained for 4 days and then returned to normal within 7–10 days. However, nonsurvivors of septic shock maintained normal left ventricular EF and volume. Similarly, Parker et al. used the same methodology and observed right ventricular dysfunction in septic shock patients. They found that the survivors and nonsurvivors of septic shock maintained a reversible reduction in biventricular EF and increased end-diastolic and -systolic volumes in the study period [21]; these results are different from those found in their previous study.

During the same historical period, two-dimensional echocardiography was performed to evaluate myocardial function in septic patients [22, 23]. Because echocardiography is a first-line non-invasive technique for hemodynamic evaluation in patients with cardiovascular disorders and it can be performed at the bedside, various echocardiographic indices, such as EF and cardiac index, have been developed to assess cardiac function. Vieillard Baron and coworkers investigated 40 patients with septic shock by transesophageal echocardiography and observed that stroke index was strongly correlated with left ventricular EF, while left ventricular volume always remained in a normal range after adequate preload optimization [24]. In 2013, a meta-analysis that included more than 700 patients failed to find any evidence to support the above view that the survivors from severe sepsis or septic shock had a reduced EF. In addition, there were no significant differences between septic survivors and nonsurvivors in terms of biventricular EF and indexed biventricular dimensions [25]. In another meta-analysis, a cut-off of left ventricular EF of 50 % was used to identify patients with systolic dysfunction, and no significant difference in mortality rates was found in septic patients with reduced EF compared to patients with normal EF [8]. Evidently, these studies revealed a complicated and contradictory picture about myocardial dysfunction in septic patients. It is now known that these inconsistent results from the above studies are very likely due to the limitations of currently used indices of ventricular function because cardiac index and EF are load-dependent indices that do not reflect the intrinsic myocardial contractile function during sepsis [16].

To detect subtle myocardial dysfunction during sepsis, some investigators examined myocardial function during sepsis using advanced echocardiographic techniques, such as speckle tracking and Doppler tissue echocardiography. Speckle tracking echocardiography is more sensitive than the conventional echocardiographic technique and is able to detect ventricular strain that reflects segmental myocardial deformation. It was demonstrated that peak left ventricular global longitudinal systolic strain, determined by using speckle tracking echocardiography at the time of admission in septic patients, correlated with mortality rate, whereas left ventricular EF had no prognostic relevance [9]. Another clinical investigation also suggested that strain imaging by speckle tracking echocardiography might be useful in the early detection of myocardial dysfunction in sepsis. It showed that 50 % of septic patients with preserved left ventricular EF had a depressed left ventricular global longitudinal function compared to non-septic patients. In patients with sepsis and preserved left ventricular EF, both left ventricular global and right ventricular free wall strain were lower than in non-septic patients with preserved left ventricular EF. These findings indicate that left ventricular and right ventricular systolic dysfunction in patients with early septic shock and preserved left ventricular EF can be detected by speckle tracking echocardiography [26]. It is noteworthy that the clinical features of segmental ventricular dysfunction during SIMD are sometimes consistent with Takotsubo cardiomyopathy, in which the contractile function of the middle-to-apical segments of the left ventricle is depressed and there is hyperkinesis of the basal walls, inducing the balloon-like appearance of the distal ventricle [27].

In contrast to systolic dysfunction, diastolic dysfunction is often ignored, and its role in determining early mortality from sepsis has not been adequately investigated. Recently, it has been demonstrated that the mitral annular early diastolic peak velocity (e’ wave) obtained by tissue Doppler imaging is one of the most load-independent measures of diastolic dysfunction. The ratio of the early mitral inflow velocity (E), recorded with pulsed-wave Doppler, to the e’ wave (E/e’) correlates with left ventricular end-diastolic pressure, and a high E/e’ ratio represents low left ventricular compliance in numerous cardiac conditions [28, 29]. Using the reduced e’ wave or the increased E/e’ ratio to identify left ventricular diastolic dysfunction, some investigators found that diastolic dysfunction was common in septic patients. A reduced mitral annular e’-wave was the strongest predictor of mortality, and E/e’ was an independent predictor of hospital survival, which offered better discrimination between hospital survivors and non-survivors than cardiac biomarkers such as cardiac-specific troponins (cTn) and N-terminal proB-type natriuretic peptide (NT-proBNP) [7, 30, 31].

### Alterations in electrocardiogram

In 1982, Terradellas et al. reported the acute elevation of the ST segment in bacterial shock patients with no history of heart disease [32]. Other investigators then found that endotoxin induced initial tachycardia followed by significant bradycardia and elevation of the ST segment in rats [33]. However, it was also reported that ST-segment elevations were rare in septic shock patients in the setting of normal coronary angiography. In addition, the electrocardiographic changes during septic shock also include a loss of QRS amplitude, increase in QT interval, development of narrowed QRS intervals with deformed bundle branch blocks [34] and new-onset atrial fibrillation, especially in older septic shock patients [35].

## Biomarkers of SIMD

B-type natriuretic peptide (BNP) is a hormone synthesized in the myocardium. It is produced in the prohormone form, and before secretion is split into the inactive NT-proBNP and the active BNP [36]. Numerous studies demonstrated markedly elevated plasma levels of BNP and NT-proBNP in septic patients [37, 38], and the increased plasma BNP and NT-proBNP concentrations were associated with myocardial depression and increased mortality in patients with sepsis [39, 40]. These findings suggest that plasma BNP and NT-proBNP levels represent reliable markers for the identification of SIMD. However, other studies indicate that the relationship between BNP and both left ventricular EF and left-sided filling pressures is weak and data on the prognostic impact of high BNP levels in septic patients are conflicting [41]. In fact, many factors, including right ventricular overload, catecholamine therapy and increased cytokine production, may contribute to BNP release during sepsis. Thus, it is suggested that cTn may be integrated into the monitoring of SIMD [41]. Plasma cTnI and cTnT levels have also been proven to be highly sensitive and specific markers of SIMD [14]. Although the mechanisms underlying cTnI release during sepsis are still unclear, increased plasma concentrations of cTnI and cTnT were found in septic patients, and both cTnI and cTnT were exclusively associated with left ventricular dysfunction [42]. A meta-analysis showed that plasma troponin elevation in septic patients was also a predictor of mortality [43]. In addition, serum heart-type fatty acid-binding protein concentration was also found to be a useful diagnostic marker for organ dysfunction and 28-day mortality in septic patients [44, 45]. Zhang et al. found that serum heart-type fatty acid-binding protein was frequently elevated in septic patients and appeared to be associated with SIMD [46]; large prospective clinical trials on its role in identifying SIMD are now warranted.

## Cardiac structural changes

Human autopsies and animal experimental studies have revealed that sepsis-induced myocardial changes are classified within inflammatory cardiomyopathy. The major cardiac pathological changes during sepsis include myocardial infiltration by immune cells (especially macrophages and neutrophils), subendocardial hemorrhage, interstitial and intracellular edema, endothelial cell edema, microcirculatory fibrin deposition, as well as focal myofibrillar dissolution, cardiomyocyte necrosis and interstitial fibrosis. Intracytoplasmic lipid accumulation in cardiomyocytes is also observed in septic hearts. Immunohistochemical examination shows that high diffuse expression of tumor necrosis factor-α (TNF–α) is localized to cardiomyocytes, macrophages, smooth muscle cells and endothelial cells [12, 47–49]. As mentioned above, SIMD is thought to be completely reversible. However, we do not know whether the histological myocardial alterations in sepsis are reversible. Therefore, the possibility that myocardial dysfunction is not completely reversible in septic patients should be further examined.

## Pathogenesis of SIMD

Based on an animal study, the first hypothesis on the mechanism of SIMD was global myocardial ischemia resulting from inadequate coronary blood flow. However, coronary blood flow was later found to be either preserved or increased in septic shock patients with myocardial dysfunction, disproving the above hypothesis. Although some studies demonstrated that impairment in cardiac microcirculation was present during sepsis due to significant maldistribution of coronary blood flow, endothelial damage, intravascular fibrin depositions and neutrophil infiltration, which might result in focal myocardial ischemia and decreased cardiac function, no myocardial hypoxia was confirmed in septic animals. It is now suggested that the elevation of plasma cardiac troponins may be attributed to an increase in cardiomyocyte membrane permeability rather than myocardial ischemic necrosis. These findings indicate that coronary circulation alterations are less important in the mechanisms of SIMD [6, 50]. According to the current evidence, it is generally accepted that SIMD may be a result of the interaction of many factors, including inflammation, metabolism and neuroimmunomodulation.

### Myocardium-depressing factors

In the 1960s, many investigators reported the presence of myocardium-depressing factors that could cause SIMD [12]. During sepsis, various pathogen-associated molecular patterns (PAMPs), such as lipopolysaccharide (LPS), and endogenous damage-associated molecular patterns (DAMPs), including high mobility group box 1 (HMGB1) and extracellular histones, interact with Toll-like receptors (TLRs) on immune cells and other cells. All TLRs, except TLR3, signal through the myeloid differentiation factor 88 (MyD88)-dependent pathway and activate c-Jun N-terminal kinase (JNK), extracellular signal-regulated kinases 1/2 (ERK1/2), p38 mitogen-activated protein kinase (MAPK) and the transcription factor nuclear factor (NF)-kB signaling pathways, which in turn induce the production of multiple proinflammatory cytokines, including interleukin (IL)-1, IL-6 and TNF-α [51, 52]. Several substances have been considered as myocardium-depressing factors, including TNF-α, IL-1, IL-6, complement anaphylatoxin (C5a) and LPS (Table 1) [12, 14]. For example, prolonged exposure of adult rat ventricular myocytes to a mixture of LPS, TNF-α, IL-1 and IL-6 inhibited cell contractility in vitro [53], and treatment with a monoclonal anti-TNF antibody in patients within 24 h of septic shock improved left ventricular function [54]. However, a recent clinical study investigated the relationship between serum cytokine concentrations (IL-1β, IL-6, IL-8, IL-10, IL-18, TNF-α and monocyte chemoattractant protein-1) and septic myocardial dysfunction. The authors defined reduced left ventricular EF of < 50 % or < 55 % as systolic dysfunction and e wave < 8 cm/s as diastolic dysfunction and found that none of these cytokines correlated with left ventricular EF and e’-wave velocities in septic patients. Similarly, there were no differences in cytokine concentrations between patients dichotomized to high and low left ventricular EF or e wave. Therefore, none of the measured circulating cytokines correlated with systolic or diastolic myocardial dysfunction in severe sepsis or septic shock in the clinical setting [55]. More recently, experimental studies found that LPS induced myocardial HMGB1 expression and increased plasma HMGB1 level in rats and mice and that HMGB1 stimulation produced a negative inotropic effect in the isolated rat heart [56, 57]. Similarly, another study demonstrated that increased circulating histone levels were significantly associated with new-onset left ventricular dysfunction and arrhythmias in septic patients with no previous cardiac dysfunction [58]. Nevertheless, the role of circulating HMGB1 and histones in SIMD deserves to be further investigated.

**Table 1:** Myocardial depressant factors in sepsis

Classification	Myocardial depressant factor
Cytokines	Interleukin-1 Interleukin-6 Tumor necrosis factor-α
Complement components	Activated complement 3 Complement anaphylatoxin (C5a)
Pathogen-associated molecular patterns	LPS
Damage-associated molecular patterns	High mobility group box 1 Extracellular histones
Matrix metalloproteinases	Matrix metalloproteinase-9

Indeed, it is likely that circulating myocardium-depressing factors are the initial stimuli and driving forces of septic myocardial dysfunction. It is well known that cardiomyocytes express Toll-like receptors such as TLR2 and TLR4 [59, 60]. LPS stimulates TLR4 on cardiomyocytes and leads to the phosphorylation of p38 MAPK and JNK and the activation of NF-kB, which induces cardiomyocyte TNF-α expression and decreases myocardial contractility [61]. Natural deletion of TLR4 [62] or MyD88 deletion in cardiomyocytes confers a profound protection with markedly improved cardiac function and survival in an LPS-induced shock model [63]. In addition, extracellular histones also stimulate cardiomyocyte TLR4 and induced myocardial dysfunction [64]. We recently observed that stimulation of cardiomyocyte β_1_-adrenoceptor promoted p38MAPK, JNK and NF-kB activation and subsequent TNF-α expression in LPS-treated cardiomyocytes [65]. Activation of cardiomyocyte α_1_-adrenoceptor can suppress LPS-induced cardiomyocyte TNF-α expression and improve cardiac dysfunction during endotoxemia [66]. We also found that blockade of the α_2_-adrenoceptor suppressed myocardial TNF-α and inducible nitric oxide synthase (iNOS) expression and cardiomyocyte apoptosis and cardiac dysfunction in endotoxemic animals [67]. Thus, it is necessary for regulating cardiomyocyte adrenergic signals to develop interventions for some myocardium-depressing factors and to provide therapeutic targets for SIMD.

In addition to cardiomyocytes, cardiac fibroblasts and endothelial cells are involved in SIMD. Cardiac fibroblasts make up 60 %–70 % of the total cell number in the heart. Tomita et al. demonstrated that LPS significantly increased the expression of TNF-α and matrix metalloproteinase (MMP)-9 in cultured cardiac fibroblasts. CLP induced cardiac MMP-9 expression, cardiac fibrosis and cardiac dysfunction in mice, and treatment with a broad-spectrum MMP inhibitor significantly alleviated these histological and functional changes during sepsis [68]. In addition, endothelial cell activation also plays a critical role in septic injury in multiple organs. It has been demonstrated that serum levels of sphingosine-1-phosphate, a potent regulator of endothelial integrity, are dramatically decreased and inversely associated with disease severity in septic patients [69]. Clinical evidence showed that systolic cardiac dysfunction was directly associated with markers of endothelial dysfunction in septic patients [70]. Some studies reported that circulating myocardium-depressing factors, such as TNF-α, increased the expression of intercellular adhesion molecule-1 (ICAM-1) and vascular cell adhesion molecule-1 (VCAM-1) in coronary endothelial cells and cardiomyocytes [71, 72]. Blockade of VCAM-1 reduced myocardial neutrophil accumulation and abrogated LPS-induced cardiac dysfunction. The absence of ICAM-1 also reduced LPS-induced cardiac dysfunction, but without decreasing neutrophil accumulation. Moreover, the depletion of neutrophils failed to protect against LPS-induced myocardial dysfunction. These results indicate that endothelial and/or cardiomyocyte ICAM-1 and VCAM-1 mediate LPS-induced myocardial dysfunction independent of neutrophil infiltration [73]. Some investigators generated double transgenic mice that conditionally overexpress a degradation-resistant form of I-kB, an inhibitor of NF-kB, selectively on the endothelium. These authors demonstrated that endothelial-selective blockade of NF-kB activation markedly inhibited cardiac ICAM-1 and VCAM-1 expression and ameliorated myocardial injury in both LPS and CLP models of sepsis [74]. Therefore, circulating myocardium-depressing factors may activate cardiac fibroblasts and endothelial cells, which contribute to SIMD. Collectively, circulating PAMPs, DAMPs and cytokines can activate endothelial cells, cardiac fibroblasts and cardiomyocytes and increase the production of inflammatory mediators, which further stimulate iNOS expression and cause myocardial depression in sepsis [12, 50, 53, 64].

### Autonomic dysregulation

The autonomic nervous system plays an important role in sepsis. Some evidence indicates that sepsis induces autonomic dysregulation, including neuronal and glial apoptosis within the autonomic centers of the heart, high plasma levels of catecholamines, reduced heart rate variability and decreased cardiac responsiveness to intrinsic catecholamines, which may contribute to SIMD. A number of studies showed decreased densities of β_1_-adrenoceptors, reduced levels of stimulatory G-proteins and increased expression of inhibitory G-proteins in cardiomyocytes during sepsis. These results indicate that impaired myocardial responsiveness to catecholamines in sepsis can be attributed to the downregulation of adrenergic receptors and/or post-receptor signaling [6, 75]. In addition, β_3_-adrenoceptors, which mediate an increased negative inotropic response to agonists, were found to be upregulated during sepsis, suggesting that activation of β_3_-adrenoceptors by catecholamines may contribute to SIMD [76].

### Dysfunction of intracellular Ca^2+^ transporters in cardiomyocytes

In addition to a decrease in myofilament Ca^2+^ sensitivity due to a sustained increase in cardiac troponin I phosphorylation at Ser23/24 [77], a dysfunction of intracellular Ca^2+^ transporters in cardiomyocytes underlies SIMD. During sepsis, downregulated L-type calcium channels and a suppressed sarcoplasmic reticulum (SR) pump lead to a decrease in the amplitude of cellular Ca^2+^ transients and SR calcium load in cardiomyocytes [78]. CLP increased myocardial angiotensin II content, which may be associated with the disturbance of Ca^2+^ transport in the cardiac SR [79]. LPS also specifically impaired sarcolemmal diastolic Ca^2+^ extrusion pathways by depressing the function of the Na^+^/Ca^2+^exchanger and the plasmalemmal Ca^2+^ ATPase, which in turn resulted in intracellular diastolic Ca^2+^ overload [80]. This disruption of cellular Ca^2+^ homeostasis in cardiomyocytes may contribute to SIMD. However, the mechanism underlying the reduction in transient systolic Ca^2+^ is not well established. A recent study demonstrated that sepsis induced a decrease in sodium current in cardiomyocytes, which reduced cardiac excitability. This reduction in the density of Na^+^ channels might lessen the transient Ca^2+^ action potential in cardiomyocytes by decreasing the number of Ca^2+^ channels that open during the action potential due to the reduction in peak depolarization or/and by reducing Ca^2+^ entry due to a shortening of the action potential [81].

### Energetic starvation of cardiomyocytes

Although oxygenation of the myocardium does not appear to be altered during sepsis, accumulating evidence indicates that impaired metabolism and reduced energy production in cardiomyocytes play a critical role in SIMD. Under normal conditions, approximately 70 % of adenosine triphosphate (ATP) in cardiomyocytes is produced via fatty acid oxidation, and the remainder is produced via glucose oxidation. A small amount of ATP is also derived from the catabolism of lactate and ketone bodies [82]. During sepsis, inflammatory cytokines, such as IL-1β, can downregulate very low-density lipoprotein receptor expression in cardiomyocytes [83]. Decreased expression of the very low-density lipoprotein receptor and the fatty acid transporter CD36 inhibits lipid uptake by cardiomyocytes [82]. Importantly, Toll-like receptor-mediated inflammatory signaling reprograms cardiac energy metabolism, leading to a reduced expression of fatty-acid-binding protein, acyl-CoA synthetase, and fatty acid oxidation-associated transcriptional factors, including peroxisome proliferator activated receptors (PPARs) and PPARγ-coactivator-1 [82, 84]. Recently, Drosatos et al. discovered that cardiomyocyte Krüppel-like factor five upregulated PPARγ expression through direct promoter binding, which was blocked in sepsis. Depletion of cardiac myocyte-specific Krüppel-like factor five not only reduced myocardial PPARγ expression, fatty acid oxidation and ATP levels, but also increased myocardial triglyceride accumulation and induced myocardial dysfunction [85]. These data indicate that sepsis inhibits intracellular fatty acid oxidation and could eventually reduce cardiomyocyte ATP production and myocardial function. Restoration of myocardial fatty acid oxidation improves SIMD. Drosatos et al. demonstrated that both cardiomyocyte-specific expression of PPARγ and activation of PPARγ by rosiglitazone increased myocardial fatty acid oxidation and prevented LPS-induced cardiac dysfunction, but without affecting the expression of myocardial inflammatory cytokines [86]. Thus, although inflammation is an important component of mechanisms that mediate SIMD, the decrease in myocardial fatty acid oxidation constitutes another critical mechanism responsible for this disorder.

### Mitochondrial dysfunction and oxidative-nitrosative stress

Although early myocardial dysfunction during sepsis is associated with myocardial inflammation rather than mitochondrial injury [87], enzyme activities of nicotinamide-adenine dinucleotid cytochrome c reductase, succinate cytochrome c reductase and cytochrome c oxidase were found to be significantly suppressed during sepsis. Mitochondrial complex II and complex IV were also downregulated, and the myocardial ATP content markedly declined during the late stage of sepsis [88]. These results indicate that mitochondrial dysfunction associated with a decrease in myocardial ATP content is likely correlated with the deterioration of myocardial function during the late stage of sepsis. Furthermore, pharmacological inhibition of mitochondrial permeability transition by cyclosporine derivatives was found to improve myocardial dysfunction and survival in animal models of CLP-induced sepsis [89]. Similarly, the administration of mitochondria-targeted vitamin E also protected myocardial mitochondrial structure and function, inhibited mitochondrial oxidative stress, and improved myocardial function in septic rats [90]. Thus, it is likely that mitochondrial dysfunction is causative rather than epiphenomenal and is relevant to SIMD. However, the underlying mechanisms responsible for sepsis-induced mitochondrial dysfunction are still not completely elucidated. Oxidative-nitrosative stress due to excessive production of mitochondrial reactive oxygen species and nitric oxide, increased mitochondrial permeability transition pore opening and increased mitochondrial uncoupling may contribute to this type of mitochondrial dysfunction [91, 92].

### Cardiomyocyte apoptosis

In addition to leading to DNA fragmentation, activated caspases can directly induce the breakdown of myofibrillar proteins, decrease ATPase activity and force development in cardiomyocytes [93]. Although cardiomyocyte apoptosis has not been observed in human autopsy specimens, there is increasing evidence that caspase-3 activation and cardiomyocyte apoptosis contribute to SIMD [94–96]. It is generally accepted that the over-production of inflammatory mediators and reactive oxygen species plays a critical role in capase-3 activation and cardiomyocyte apoptosis during sepsis [95, 97–99]. However, we recently found that reduced cardiac endogenous norepinephrine or blockade of β_1_-adrenoceptors almost completely abolished cardiomyocyte apoptosis in LPS-challenged mice [67]. We further demonstrated that β_1_-adrenoceptor activation promotes LPS-induced cardiomyocyte apoptosis [65]. Therefore, β_1_-adrenoceptor activation appears to be more important than cytokines in LPS-induced cardiomyocyte apoptosis. In fact, a randomized clinical trial observed that therapy with the β-blocker esmolol reduced heart rate, increased left ventricular stroke work index and decreased 28-day mortality in septic shock patients [100]. The reader can refer to the excellent systematic review on β-blockers in septic patients [101].

## Conclusions

SIMD refers to the intrinsic myocardial systolic and diastolic dysfunction of both the left and right sides of the heart during sepsis. Early recognition of intrinsic myocardial dysfunction is critical for the administration of the most appropriate therapy for septic patients. However, traditional insensitive parameters, such as EF, cannot accurately assess sepsis-induced intrinsic myocardial dysfunction. It is now suggested that the routine use of speckle tracking and tissue Doppler echocardiography may be valuable in the identification of SIMD in septic patients. The circulating myocardium-depressing factors are only initial stimulators that induce cardiac structure and function damage, in which cardiomyocytes, cardiac endothelial cells and even fibroblasts are involved. A deeper understanding of the effects of immuno-metabolic and neuroendocrine factors on cardiomyocytes, endothelial cells and fibroblasts at molecular and subcellular levels will expand our knowledge of the mechanisms contributing to SIMD. The elucidation of these mechanisms should help identify new cardiac-specific therapeutic targets and improve the prognosis of septic patients.

## Abbreviations

BNP: B-type natriuretic peptide
CLP: Cecal ligation and puncture
CO: Cardiac output
cTn: cardiac-specific troponins
DAMPs: Damage-associated molecular patterns
EF: Ejection fraction
ERK1/2: Extracellular signal-regulated kinases 1/2
HMGB1: High mobility group box 1
ICAM-1: Intercellular adhesion molecule-1
iNOS: inducible nitric oxide synthase
JNK: c-Jun N-terminal kinase
LPS: Lipopolysaccharide
MAPK: Mitogen-activated protein kinase
MMP: Matrix metalloproteinase
MyD88: Myeloid differentiation factor 88
NF-kB: Nuclear factor-kB
PAMPs: Pathogen-associated molecular patterns
PPARs: Peroxisome proliferator activated receptors
SIMD: Sepsis-induced myocardial dysfunction
SR: Sarcoplasmic reticulum
SVR: Systemic vascular resistance
TNF-α: Tumor necrosis factor-α
TLRs: Toll-like receptors
VCAM-1: Vascular cell adhesion molecule-1
